# A Rare Case of Cerebral Venous Sinus Thrombosis Following the Second Dose of BNT162b2 mRNA COVID-19 Vaccine—Just a Coincidence? A Case Report

**DOI:** 10.3390/reports8020050

**Published:** 2025-04-16

**Authors:** David Matyáš, Roman Herzig, Libor Šimůnek, Mohamed Abuhajar

**Affiliations:** 1 Department of Neurology, Comprehensive Stroke Centre, University Hospital Hradec Králové, CZ-500 05 Hradec Králové, Czech Republic; david.matyas@fnhk.cz (D.M.); libor.simunek@fnhk.cz (L.Š.); 2 Department of Neurology, Faculty of Medicine in Hradec Králové, Charles University, CZ-500 03 Hradec Králové, Czech Republic; 3 Department of Radiology, Comprehensive Stroke Centre, University Hospital Hradec Králové, CZ-500 05 Hradec Králové, Czech Republic; mohamed.abuhajar@fnhk.cz; 4 Department of Radiology, Faculty of Medicine in Hradec Králové, Charles University, CZ-500 03 Hradec Králové, Czech Republic

**Keywords:** COVID-19, vaccination, mRNA vaccine, cerebral venous sinus thrombosis, case report

## Abstract

**Background and Clinical Significance:** The occurrence of cerebral venous sinus thrombosis (CVST), both with or without thrombocytopenia, following COVID-19 vaccination, is well documented and more common in recipients of vector vaccines. Cases of CVST following immunization with the COVID-19 messenger RNA (mRNA) vaccine are rare; most of these cases occur within 28 days of the first dose of the vaccine. **Case Presentation:** We present the case of a 38-year-old male with a history of two episodes of deep vein thrombosis in the lower limbs, but without a specific thrombophilic condition, who developed CVST 13 days after the second dose of the Pfizer/BioNTech BNT162b2 vaccine. He suffered from diffuse tension-type headache of progressively increasing intensity, and his objective neurological findings were normal. Magnetic resonance venography showed thrombosis of the transverse and right sigmoid sinuses, and magnetic resonance imaging (MRI) of the brain revealed no cerebral infarction. Two months later, a follow-up MR venography showed partial recanalization of the affected sinuses, and a brain MRI showed no infarction. **Conclusions:** Given the temporal sequence and the absence of other possible causes, we speculate that the second dose of the COVID-19 BNT162b2 vaccine may have triggered the development of CVST.

## 1. Introduction and Clinical Significance

Cerebral venous sinus thrombosis (CVST) is a relatively rare cause of stroke, a potentially life-threatening cerebrovascular disease that accounts for approximately 0.5% of all strokes. It has a remarkable range of symptoms and manifestations, the most common of which are headache, seizures, focal neurological deficits, papilledema, and alterations in general health and consciousness [1]. The occurrence of CVST, both with and without thrombocytopenia, following COVID-19 vaccination, is well documented. This complication is more common in recipients of vector vaccines (particularly Oxford–AstraZeneca ChAdOx1 nCoV-19) than in the unvaccinated population or recipients of messenger RNA (mRNA) vaccines [2,3,4,5,6]. Cases of CVST following vaccination with the mRNA COVID-19 vaccine (Pfizer/BioNTech BNT162b2 and Moderna mRNA-1273) are rare and most usually occur within 28 days of the first vaccine dose [5,7].

Most patients with CVST following vaccination with the COVID-19 vaccine were discharged in good clinical condition, while 37% died, mostly from brain injury [3]. Therefore, a better understanding of the mechanism by which mRNA vaccination can cause CVST is needed so that vaccination can be tailored to reduce the risk of this potentially life-threatening complication. Here, we present the case of a patient with no known thrombophilic condition, who developed CVST 13 days after the second dose of the BNT162b2 vaccine.

## 2. Case Presentation

A 37-year-old male received two doses of the BNT162b2 mRNA vaccine approximately 3 weeks apart, as prescribed. Thirteen days after the second dose, he developed a diffuse tension-type headache of progressively increasing intensity (especially at night), with limited response to metamizole. Five days later, he was examined by a neurologist in the emergency department. He had a history of two episodes of deep vein thrombosis (DVT), affecting the left peroneal and tibial veins (in June 2019) and the right peroneal vein (in September 2019). Despite extensive laboratory screening (Table 1), there was no evidence of congenital or acquired thrombophilia. The results of cancer screening tests (chest X-ray, abdominal and thyroid ultrasound, urological examination, PSA, HCG, and AFP) were also negative. In addition, he had no other major illnesses and denied any family history of thrombosis. Neurological examination revealed no focal brain lesion, and the patient showed no signs of meningeal irritation.

Test kits and testing methods:

Factor V Leiden Thrombophilia—gb HEMO FV (G1691A) by Generi Biotech (Hradec Králové, Czech Republic), performed on a Rotor Gene 6000 machine by Corbett Life Science (Mortlake, Australia)/Qiagen (Hilden, Germany).

FII2010a mutation—gb HEMO FII (G20210A) by Generi Biotech (Hradec Králové, Czech Republic), performed on a Rotor Gene 6000 machine by Corbett Life Science (Mortlake, Australia)/Qiagen (Hilden, Germany).

Protein C deficiency—spectrophotometry: Biophen Protein C 5 by Hyphen BioMed (Seoul, South Korea); if activity is low, protein C antigen is measured with the ELISA: Asserachrom Protein C set by Stago (Asnières-sur-Seine, France).

Protein S deficiency—coagulation method: STA-Staclot Protein S set by Stago (Asnières-sur-Seine, France); if activity is low, free PS and total PS antigens are measured through the ELISA method: Asserachrom Free Protein S and Asserachrom Total Protein S sets by Stago (Asnières-sur-Seine, France).

Primary antiphospholipid syndrome—PTT LA by Stago (Asnières-sur-Seine, France); STA Staclot dRVV Screen 2 by Stago (Asnières-sur-Seine, France); Pool Norm by Stago (Asnières-sur-Seine, France); STA Staclot dRVVT Confirm by Stago (Asnières-sur-Seine, France); APTT-LA: STACLOT LA by Stago (Asnières-sur-Seine, France).

Seronegative antiphospholipid syndrome—clinically assessed by a hematologist.

Anti-platelet factor 4—anti-PF4 IgG—ELISA: Zimutest HIA MonoStrip IgG by Hyphen Biomed (Neuville-sur-Oise, France).

JAK-2 mutation—allelic discrimination method using qPCR, performed on a Rotor-Gene Corbett 6000 2-plex by Corbett Life Science (Mortlake, Australia)/Qiagen (Hilden, Germany), using LNA probes (in-house method).

Calreticulin—HRM real-time PCR method (in-house method), performed on a Rotor Gene Q HRM1 instrument by Corbett Life Science (Mortlake, Australia)/Qiagen (Hilden, Germany).

Antithrombin deficiency—spectrophotometry: STA-Stachrom AT III set by Stago (Asnières-sur-Seine, France), performed on a coagulometer STA R Evolution by Stago (Asnières-sur-Seine, France).

Myeloproliferative disease was excluded based on repeated normal blood count, including differential.

Monoclonal gammopathy—Hydragel 54 Protein(E) by Sebia (Lisses, France); performed on Hydrasys 2 Scan Focusing by Sebia (Lisses, France).

Paroxysmal nocturnal hemoglobinuria or another intravascular hemolysis—flow cytometry was performed on a BD FACSCANTO II machine by BD Biosciences (Franklin Lakes, NJ, USA).

Sticky platelet syndrome—Light transmission aggregometry (LTA) method with ADP and Epinephrine by Hyphen Biomed (Neuville-sur-Oise, France).

Hyperhomocysteinemia—Immulite 2000 Xpi by Siemens Healthcare (Erlangen, Germany).

Systemic vasculitis—ALEX: ALEX (Allergy Explorer) kit by BioVendor (Heidelberg, Germany); ANCA ethanol IF: NOVA LiteTM ANCA by INOVA Diagnostics, Inc. (Friedrichshafen, Germany); ANCA MPO: Anti-Myeloperoxidasis ELISA (IgG) by Euroimmun (Lübeck, Germany); ANCA PR3: Anti-PR3-hn-hr ELISA (IgG) by Euroimmun (Lübeck, Germany); ANCA profile: ANCA Profile ELISA (IgG) by Euroimmun (Lübeck, Germany); ANF: NOVA Lite HEp-2 ANA Kits/Substrate Slides by INOVA Diagnostics, Inc. (Friedrichshafen, Germany); C1 inhibitor: Human C1 Inactivator by The Binding Site Ltd. (Birmingham, UK); C4: N Antiserum to Human C4 by Siemens Healthcare Diagnostics Products GmbH (Erlangen, Germany); Complement pathway: WieslabTM Complement system Screen by SVAR (Malmö, Sweden); DAO: IDK DAO ELISA by Immundiagnostik AG (Bensheim, Germany); ECP: ECP fast by BDL Labordiagnostik GmbH (Münster, Germany); ENA screening: ANA—8S Aeskulisa by AESKU Diagnostics (Wendelsheim, Germany); FR screen: N Latex RF Kit by Siemens Healthcare Diagnostics Products GmbH (Erlangen, Germany); IgA: N Antiserum to Human IgA by Siemens Healthcare Diagnostics Products GmbH (Erlangen, Germany); IgE: Immulite 2000 total IgE by Siemens Healthineers Headquarters (Erlangen, Germany); IgG: N Antiserum to Human IgG by Siemens Healthcare Diagnostics Products GmbH (Erlangen, Germany); IgG1: N AS IgG1 by Siemens Healthineers Headquarters (Erlangen, Germany); IgG2: N AS IgG2 by Siemens Healthineers Headquarters (Erlangen, Germany); IgG3: N Latex IgG3 by Siemens Healthineers Headquarters (Erlangen, Germany); IgG4: N Latex IgG4 by Siemens Healthineers Headquarters (Erlangen, Germany); IgM: N Antiserum to Human IgM by Siemens Healthcare Diagnostics Products GmbH (Erlangen, Germany); Tryptasis: ImmunoCAP™ Tryptase by Phadia AB (Uppsala, Sweden).

Basic biochemical, hematological, and coagulation tests showed no significant abnormalities except for slightly elevated D-dimer and leukocyte levels (Table 2).

**Figure 1:**
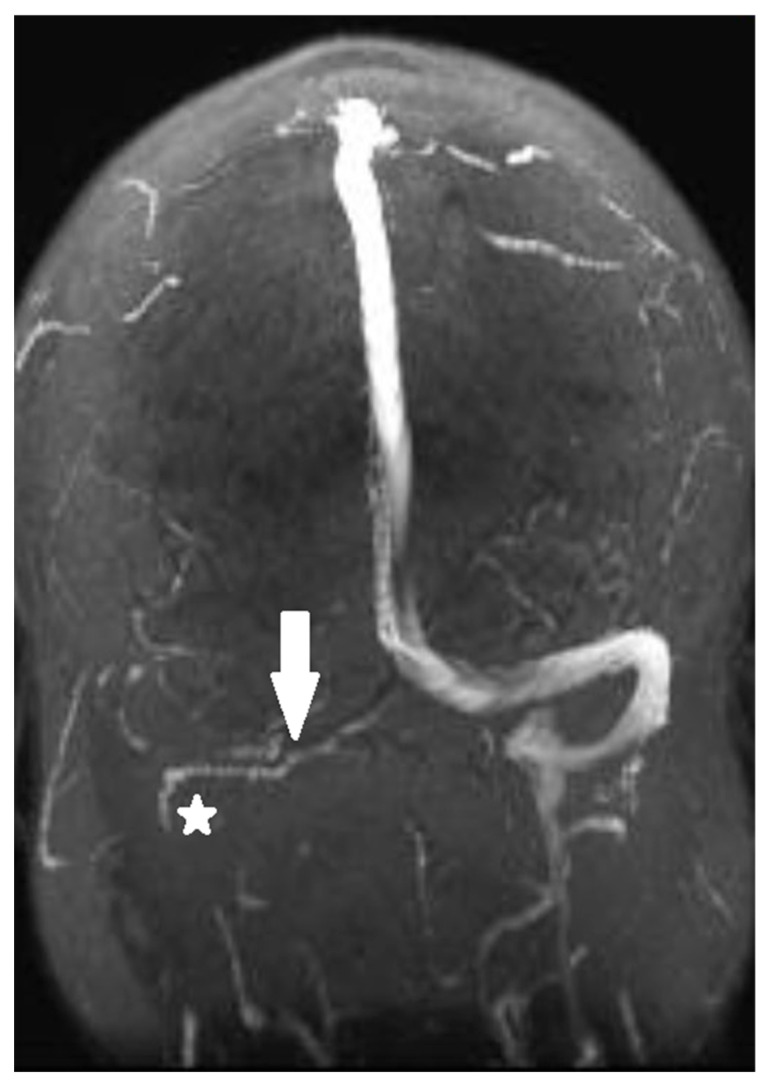
Magnetic resonance venography shows thrombosis of the transverse (arrow) and right sigmoid (asterisk) sinuses.

**Figure 2:**
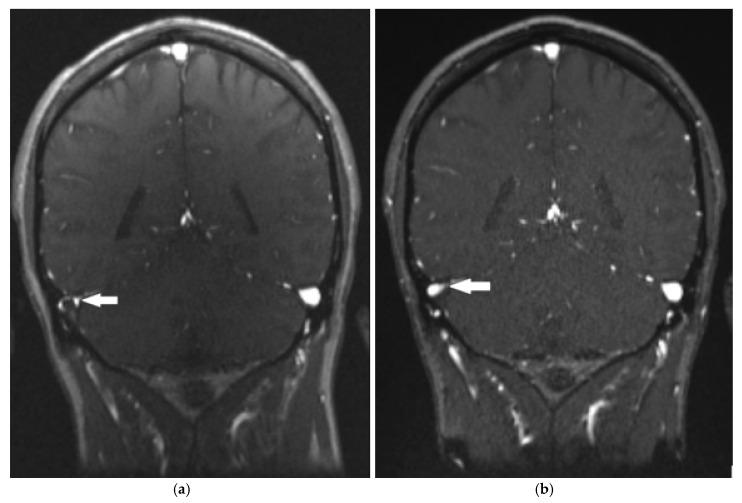
Magnetic resonance venography shows thrombosis of the transverse sinus (**a**) and its partial recanalization after two months (**b**) (arrows).

Magnetic resonance venography performed in the emergency department showed thrombosis of the transverse and right sigmoid sinuses, as shown in Figure 1 and Figure 2a. The thrombus was isointense to the gray matter on the T1-weighted image (T1WI) and slightly hyperintense to the gray matter on the T2-weighted image (T2WI). No cerebral infarction was detected on brain magnetic resonance imaging (MRI).

The patient was admitted to the neurology department, and treatment with low molecular weight heparin (nadroparin twice daily in a dose adjusted to the body weight, i.e., 2 × 7600 IU/day) was started. Throughout the 6-day hospital stay, his objective neurological findings were normal, and he was discharged home with a persistent headache of minimal intensity. Since discharge, the patient has been taking dabigatran for secondary prevention. Repeated blood samples showed no evidence of thrombocytopenia. Two months later, a follow-up MR venography showed partial recanalization of the affected sinuses (Figure 2b); a brain MRI showed no infarction, and the patient denied having any headaches. The same results were obtained seven months after the onset of symptoms.

## 3. Discussion

The estimated incidence of CVST with thrombocytopenia is 0.1 per million persons per month [5]. While an increased incidence of thrombotic events (including CVST) has been reported following adenovirus vector-based vaccination, fewer CVSTs have been reported following mRNA-based vaccines [8], and opinions on whether COVID-19 mRNA vaccines increase the risk of CVST (both with and without thrombocytopenia) are controversial. For example, based on data from the European Medicines Agency’s EudraVigilance database, Krzywicka et al. reported that within 28 days of the first vaccine dose, recipients of mRNA vaccines were not at increased risk of CVST with thrombocytopenia, whereas the absolute risk of CVST was 0.6 per million first doses for both BNT162b2 and mRNA-1273 vaccines [5]. Kerr et al. also found no increased risk of CVST following vaccination with the BNT162b2 vaccine in a pooled, self-controlled case series study of 11.6 million people in England, Scotland, and Wales [4]. Joy et al. found no associations between increased risk of thrombocytopenic, thromboembolic, and hemorrhagic events post-vaccination with the second dose of the BNT162B2 vaccine [9]. In addition, Gil-Díaz et al. found no thrombotic recurrences within 30 days of vaccination with the COVID-19 vaccine in 62 patients with a history of previous CVST, 43 of whom received the BNT162b2 vaccine [10]. Although the work of Chemaitelly et al. investigating the risk of different types of stroke following mRNA vaccination in Qatar suggested an association between CVST and vaccination, their results were not statistically significant [11]. On the other hand, Hippisley-Cox et al. found an increased risk of CVST 15–21 days after the first dose of the BNT162b2 vaccine (incidence rate ratio 3.58, 95% confidence interval 1.39 to 9.27) in an analysis of data from approximately 30 million people vaccinated against COVID-19 in England [12]. Faksova et al. also found an increased risk of CVST in BNT162b2 vaccine recipients up to 42 days after vaccination in the study, in which a total of 183,559,462 doses of the BNT162b2 vaccine were administered (observed versus expected ratio of CVST 1.49, 95% confidence interval 1.26 to 1.75 after the first dose, and 1.25, 95% confidence interval 1.06 to 1.46 after the second dose) [6].

Cases of CVST occurring after the second dose of the BNT162b2 vaccine are very rare [13,14,15]. In our patient, CVST occurred 13 days after the second dose of this vaccine. On the MRI performed five days after the onset of headache when the patient was seen by a neurologist (and at the same time 18 days after vaccination), the thrombus was isointense to the gray matter on the T1WI. This is typical for the acute phase of the thrombosis (within 7 days) due to paramagnetic deoxyhemoglobin in the red blood cells trapped within the thrombus. In the acute phase, the thrombus is usually hypointense on T2WI [16]. Nevertheless, in our patient, the thrombus was slightly hyperintense to the gray matter on the T2WI. Thrombus that is isointense on T1WI and hyperintense on T2WI is usually found in the chronic stage (after 15 days), and these intensities are probably related to the vascularized connective tissue of chronic thrombus. However, significant variability can be observed in the appearance of each stage of thrombosis in different segments in a particular patient, as well as between patients at the same clinical stage [16]. In our patient, a slight thrombus hyperintensity on T2WI could also be caused by the hypoplasia of the transverse sinus.

The localization of the thrombosis in the transverse and right sigmoid sinuses is in agreement with the results reported by de Gregorio et al., who found thrombi in the transverse sinus in 84% and the sigmoid sinus in 66% of 552 patients with CVST after COVID-19 vaccination (mostly after vector vaccines). Most (63%) of these patients were discharged in good clinical condition, sometimes with various neurological sequelae, while 37% died, largely due to brain injury (mainly intracranial bleeding) [3]. Our patient suffered no brain tissue damage and was discharged home with a persistent headache of minimal intensity and normal objective neurological findings.

Thrombosis with thrombocytopenia syndrome (TTS), which develops after administration of an adenovirus vector-based vaccine, is mediated by platelet-activating antibodies to platelet factor 4 (PF4). It clinically mimics heparin-induced thrombocytopenia (HIT). HIT is caused by platelet-activating antibodies that recognize multimolecular complexes between PF4 and heparin. TTS antibodies bind to a site on PF4 similar to that of HIT antibodies and form immune complexes that subsequently lead to the activation of platelets [17,18,19]. The mRNA-based vaccine, on the other hand, contains mRNA particles that bind to antigen recognition receptors. This initiates a pro-inflammatory cascade [20,21] with the activation of the complement, which subsequently triggers endotheliopathy with the release of ultra-large von Willebrand factor and platelet activation, leading to venous microthrombosis; these microthrombi then encounter fibrin meshes (from non-vaccine-related vascular injury, in the case of CVST an unreported head injury—a common outpatient accident), leading to venous combined micro- and macrothrombosis [22].

Although there is a clear temporal association between COVID-19 vaccination and CVST in our case, it is important to remember that temporal association does not imply causation. The development of CVST may have been purely due to the patient’s presumed thrombophilic state, and the temporal association with COVID-19 vaccination may have been coincidental. However, the thrombophilic state is only suspected because of the patient’s history of two unexplained episodes of DVT. Despite extensive investigations for thrombophilic conditions in the past, none were found. We must therefore consider the possibility that the second dose of the COVID-19 BNT162b2 vaccine may have been the triggering factor for the development of CVST.

## 4. Conclusions

In conclusion, we describe a patient who developed CVST following the second dose of the COVID-19 BNT162b2 vaccine. The available data regarding CVST after the second dose of the COVID-19 mRNA vaccine are often conflicting, and in this case, the development of CVST may have been purely due to the patient’s presumed thrombophilic condition, with the temporal association with BNT162b2 vaccination being purely coincidental. Nevertheless, physicians should be aware of this potential, albeit rare, life-threatening complication, which may occur following vaccination with COVID-19 mRNA vaccines, even after the second dose.

## Figures and Tables

**Table 1:** Examined thrombophilic conditions.

Examined Condition	Result
Factor V Leiden Thrombophilia	Negative
FII2010a mutation	Negative
Protein C deficiency	Negative
Protein S deficiency	Negative
Primary antiphospholipid syndrome	Negative
Seronegative antiphospholipid syndrome	Negative
Anti-platelet factor 4	Negative
JAK-2 mutation	Negative
Calreticulin	Negative
Antithrombin deficiency	Negative
Myeloproliferative disease	Negative
Monoclonal gammopathy	Negative
Paroxysmal nocturnal hemoglobinuria or another intravascular hemolysis	Negative
Sticky platelet syndrome	Negative
Hyperhomocysteinemia	Negative
Systemic vasculitis	Negative

**Table 2:** Selected laboratory results.

Laboratory Data	Value
Leukocytes	11.97 × 10^9^/L
Erythrocytes	5.06 × 10^9^/L
Hemoglobin	160 g/L
Thrombocytes	205 × 10^9^/L
D-dimers	0.83 mg/L
INR	0.81
APTT	31.5 s
Creatinine	84 umol/L
CRP	1.9 mg/L

## Data Availability

The data presented in this study are available upon request from the corresponding author. Due to privacy restrictions, they are not publicly available.
